# VEGF165 induces differentiation of hair follicle stem cells into endothelial cells and plays a role in *in vivo* angiogenesis

**DOI:** 10.1111/jcmm.13089

**Published:** 2017-02-28

**Authors:** Renfu Quan, Weibin Du, Xuan Zheng, Shichao Xu, Qiang Li, Xing Ji, Ximei Wu, Rongxue Shao, Disheng Yang

**Affiliations:** ^1^ Research Institute of Orthopedics The Affiliated JiangNan Hospital of Zhejiang Chinese Medical University Hangzhou China; ^2^ Department of Pharmacology School of Medical Zhejiang University Hangzhou China; ^3^ Research Institute of Orthopedics Zhejiang Chinese Medical University Hangzhou Zhejiang Province China; ^4^ Research Institute of Orthopedics The Second Affiliated Hospital School of Medical Zhejiang University Hangzhou China

**Keywords:** hair follicle stem cells, vascular endothelial cells, VEGF165, inducible factor, tissue engineering

## Abstract

Within the vascular endothelial growth factor (VEGF) family of five subtypes, VEGF165 secreted by endothelial cells has been identified to be the most active and widely distributed factor that plays a vital role in courses of angiogenesis, vascularization and mesenchymal cell differentiation. Hair follicle stem cells (HFSCs) can be harvested from the bulge region of the outer root sheath of the hair follicle and are adult stem cells that have multi‐directional differentiation potential. Although the research on differentiation of stem cells (such as fat stem cells and bone marrow mesenchymal stem cells) to the endothelial cells has been extensive, but the various mechanisms and functional forms are unclear. In particular, study on HFSCs’ directional differentiation into vascular endothelial cells using VEGF165 has not been reported. In this study, VEGF165 was used as induction factor to induce the differentiation from HFSCs into vascular endothelial cells, and the results showed that Notch signalling pathway might affect the differentiation efficiency of vascular endothelial cells. In addition, the *in vivo* transplantation experiment provided that HFSCs could promote angiogenesis, and the main function is to accelerate host‐derived neovascularization. Therefore, HFSCs could be considered as an ideal cell source for vascular tissue engineering and cell transplantation in the treatment of ischaemic diseases.

## Introduction

Tissue renewal, regeneration and repair, as well as tumorigenesis and other physiopathological processes are dominated by the occurrence of angiogenesis and vascular formation. As a key component of the vascular tissue barrier, vascular endothelial cells (VECs) are involved in these two processes, so have therefore become a favoured cell source for the construction of tissue‐engineering vascular autografts 1, 2. Owing to limited sources of autologous endothelial cells, however, patients undergoing this procedure are susceptible to graft stenosis and occlusion, resulting in exacerbation on injury. Moreover, primary VECs that have been isolated and cultured have many disadvantages including a short cell‐cycle length, limited proliferative capacity and susceptibility to ageing 3, 4, 5, 6. The acquirement of endothelial cells and promotion of early vascularization are closely correlated to wound healing and related conditions 7, 8. The use of stem cell technologies therefore might represent a major breakthrough to resolve these challenges. Stem cells can be differentiated into target cells efficiently and directly, using techniques based on induced stem cells. Thus, this area has been increasingly researched in the field of regenerative medicine 9, 10. As a class of undifferentiated and self‐renewing stem cells with strong proliferation ability *in vitro*, HFSCs can be harvested from the bulge region of the outer root sheath of the hair follicle. HFSCs have multi‐differentiation potentials, including formation of hair follicle tissues, nerve cells 11, 12, neurons and schwann cells 13, 14, 15, 16, 17, melanocytes and epidermis cells 18, blood vessel cells 19, 20, vascular smooth muscle cells and connective tissues 21, 22, and cardiac muscle cells 23, 24. Furthermore, they are available in large quantity and can be harvested with ease in autologous biopsy, without inducing serious complications 25, 26. Bassino *et al*. used transwell‐based co‐cultures to show that human follicle dermal papilla cells (FDPC) promote survival, proliferation and tubulogenesis of human micro VECs more efficiently than fibroblasts. The FDPC enhance the endothelial release of VEGF and IGF‐1, two well‐known proangiogenic growth factors 27. As a member of the VEGF family composed of five subtypes, VEGF165 has been identified to be the most active and widely distributed factor secreted by endothelial cells that play a vital role in courses of angiogenesis, vascularization and differentiation 28, 29. The Notch signalling pathway is involved in cell proliferation, differentiation, apoptosis, migration and angiogenesis and in mediating cell–cell interactions. It is therefore considered to be a highly conserved cell signalling system 30, 31, 32, 33. The γ‐secretase inhibitor (DAPT) is a specific inhibitor against the Notch signalling pathway, and this compound has been widely used in research of the transduction mechanism of the Notch signalling pathway 34, 35, 36.

Currently, research into the roles of the Notch signalling pathway in endothelial cell differentiation, proliferation, angiogenesis and neovascularization in tumour mass has been growing 37, 38. For stem cells, however, and in particular HFSCs induced to differentiate into endothelial cells, roles of the Notch signalling pathway have not been reported as yet. As shown by Western blot, DAPT successfully blocked the Notch pathway, resulting in a significant decrease in CD31 and VE‐cadherin in endothelial cells. These proteins play positive roles in promoting vascular endothelial regeneration, maintaining the vascular endothelial integrity and regulating vascular tone 39, 40. In this study, a modified *in vitro* culture method was developed based on rat HFSCs (rHFSCs) to yield suitable seed cells, and VEGF165 was used as the inducible factor for directed endothelial cells induction *in vitro*. The aim was to establish a simple and reliable method of targeted differentiation of rHFSCs into vascular ECs. DAPT was utilized to suppress the Notch signalling pathway to investigate its effect on the efficiency of inducible differentiation of rHFSCs into ECs. Finally, the *in vivo* proangiogenic feasibility of this approach was validated to clarify the role of VEGF165 in the process of vascularization *in vivo* and to generate suitable seed cells for vascular tissue engineering and cell transplantation for the treatment of ischaemic diseases.

## Materials and methods

### Experimental animals

Six clean‐grade 1‐week‐old Sprague Dawley (SD) rats weighing (24 ± 4 g, male and female) were supplied by the Laboratory Animal Center of Zhejiang province with Certificate No. SCXK (Zhejiang) 2014‐0001. Twelve 6‐week‐old male nude mice were supplied by Zhejiang University Laboratory Animal Center (ZJULAC). The conduct of animal purchase, care and disposal met all requirements of the Guide for the Care and Use of Laboratory Animals (version 2006) developed and released by the National Ministry of Science and Technology of China PR.

### Reagents

The main reagents included: knockout serum replacement (KSR), type IV collagenase, dispase enzyme, a Coating Matrix Kit (Gibco, Grand Island, NY, USA); recombinant human epidermal growth factor (EGF) and recombinant human basic fibroblast growth factor (bFGF; R&D, Minneapolis, MN, USA); type IV collagen (BD, Franklin lakes, NJ, USA); integrin‐β1 antibodies (Biolegend, San Diego, CA, USA); integrin‐α6 and VE‐cadherin antibodies (Santa Cruz Biotechnology, Inc. Shanghai, China); keratin‐15, p63 and CD31 antibodies (Abcam, Cambridge, England); 4′,6‐diamidino‐2‐phenylindole (DAPI; Roche, Bayer leverkusen, Germany); recombinant rat VEGF165 (Peprotech, Rocky Hill, NJ, USA); foetal bovine serum (FBS; Gibco, Grand Island, NY, USA); Matrigel glue (Corning, Corning, NY, USA); γ‐secretase inhibitor (DAPT; Selleck, Rocky Hill, NJ, USA); Dil‐ac‐LDL (Yiyuan Biotech, Guangzhou, China); and Texas red dextran (Invitrogen Biotech, Carlsbad, CA, USA). All primer syntheses are shown in Table 1 (Nisann Biological Technology Inc., Shanghai, China).

**Table 1:** Primers for polymerase chain reaction analysis

Gene	Primers sequences(5′–3′)	Fragment size (bp)
CD31	Forward: GAAATGGTGCTTCGGTGCTC	108
	Reverse: GGTGTCATTCACGGTTTCTTCG	
VE‐cadherin	Forward: ATGAGGTCGGTGCCCGTATT	138
	Reverse: CGTTGGTCTTGGGGTCTGTGA	
GAPDH	Forward: TGCTATGTTGCCCTAGACTTCG	240
	Reverse: GTTGGCATAGAGGTCTTTACGG	

### Isolation, culture, purification and identification of rHFSCs

#### Isolation, culture and purification of rHFSCs

The following method was modified from a previously published method 37, 38, 39. One‐week‐old SD rats were sacrificed and disinfected with 75% ethanol to yield the tentacles skin. Tissues were then washed with phosphate‐buffered saline (PBS), followed by digestion with 1% IV collagenase enzyme and 1% dispase mixture for treatment of the basement membrane matrix. The bulge region was then isolated under stereomicroscope, after demyelinating, and tissue adhesion was achieved in 10 cm Petri dish with 1 ml of complete medium (containing 869 μl/ml DMEM / F12, 100 μl/ml KSR, 10 μl/ml penicillin and streptomycin mixture, 10 μl/ml L‐glutamine, 10 μl/ml non‐essential amino acids, 20 ng/ml EGF, 10 ng/ml bFGF, 1 μl/ml polyhydric alcohol and 10 ng/ml hydrocortisone). The preparation was cultured at 37°C/5% CO_2_ for 1 hr, prior to slowly adding 2 ml of complete medium, then the preparation was cultured for another 3 hrs until tissue adherence. Then, 3 ml of complete medium was slowly added and the culture medium was changed every 2–3 days. After 8–10 days, the resultant culture was washed three times with protease (0.25% trypsin + 0.02% EDTA) diluted in PBS solution (1:3). The protease solution was then switched to TrypLE™ Select Trypsin (1×), and the preparation was treated at 37°C/5% CO_2_ for approximately 8 min. A method described by Lv *et al*. 40, 41 was also modified. In accordance with the differential adherence method of type IV collagen, type IV collagen was pre‐coated under room temperature for 1 hrs. All adherent cells within 15–20 min. were further cultured in complete medium. Cell morphology was then examined under an inverted phase contrast microscope.

#### Flow cytometry and immunofluorescence analysis

Third passage cells were harvested, and the cell density was adjusted to 1.0 × 10^6^ cells per ml. PBS was added, and the cells were washed twice by centrifugation. The resultant cells were fixed with 80% methanol under ambient condition for 5 min., after which 0.1% PBS‐Tween (PBST) was added and the resultant preparation was kept under ambient condition for 20 min., prior to the addition of 5% BSA‐PBS blocking solution with agitation for 30 min. The resultant preparation was washed once with PBS. For each tube, 100 μl (1 × annexin‐binding buffer) was added for flow cytometry with 1 × 10^6^ cells. Integrin β1‐PE, integrin‐α6, CK15, p63 and VE‐cadherin antibodies were added, and the resultant preparation was cultured in the dark for 30 min. Fluorescein‐labelled goat anti‐mouse and rabbit anti‐mouse secondary antibodies were then added, and the preparation was cultured in the dark for 30 min. and washed once with PBS. For each flow cytometry tube, 500 μl of PBS was added for a single cell suspension. The resultant preparation was subjected to flow cytometry. Negative isotype controls were used for each tube.

Similarly, third passage cells were utilized for growing cells on coverslips. After being cultured at 37°C/5% CO_2_ for 2 days, the culture medium was removed. Cells were fixed in 4% PFA‐PBS for 10 min., followed by blocking in 5% BSA‐PBS under ambient conditions. The resultant preparation was washed three times with PBS for 5 min. each wash. Integrin‐β1, integrin‐α6, CK15, P63, CD31 (1:100) and VE‐cadherin (1:50) primary antibodies were then added. For the negative control group, the primary antibody was replaced with PBST to exclude any non‐specific binding of secondary antibody. The preparation was then incubated at 4°C overnight. After the washing procedure, fluorescein‐labelled goat anti‐mouse and rabbit anti‐mouse secondary antibodies were added and the preparation was treated in the dark for 30 min. DAPI (1:2000) was added for 10 min. for nuclei staining. The final plate was mounted after drying in the dark and photographed under a laser scanning confocal microscope.

### Inducible differentiation of rHFSCs into VECs *in vitro* and related identification

#### Inducible differentiation into endothelial cells and morphology examination

VEGF165 was diluted to a working concentration of 10 ng/ml, and the KSR previously used was replaced with FBS. Other components of the medium were unchanged. Third passage rHFSCs were harvested and isolated. Step‐wise inducible *in vitro* differentiation was initiated when cells reached approximately 60% confluence. During the course of inducible differentiation, the original medium was changed to a medium containing 10 ng/ml VEGF165. Cells were then cultured at 37°C/5% CO_2_, with medium changes every 2 days. Any morphological cell changes were examined under an inverted phase contrast microscope, and photographs were taken after 1 week.

#### Expression profiles of CD31 and VE‐cadherin by flow cytometry and immunofluorescence

Cells were harvested after induction for 1 week. The detailed procedures for flow cytometry and immunofluorescence are described above.

#### Identification of WPBs by TEM

After 1 week of induction, highly efficient cells were identified and fixed with 2.5% glutaric PBS for 4 hrs or overnight. Cells were then rinsed with 0.1 M PBS, fixed with 1% osmium tetroxide, rinsed with ddH_2_O, fixed/stained with 2% uranyl acetate and gradient dehydrated in 50%, 70%, 90%, 100% ethanol and acetone, prior to infiltration, embedding and polymerization. Finally, sections were cut using an ultramicrotome, followed by staining with uranyl acetate and lead citrate. The internal structure of post‐induced cells was further examined by TEM.

#### Endothelial cell tube formation assay

Matrigel was defrosted overnight in a 4°C refrigerator. Before experimentation, pipette tips and 24‐well plates were pre‐chilled in a 4°C refrigerator for 30 min. All procedures were conducted on ice. Matrigel at a concentration of 50 μl/cm^2^ was added to one well of the 24‐well plate and was then homogenized with gentle agitation. The preparation was transferred to a 37°C/5% CO_2_ incubator for 30 min. to form a gel. After 1 week, cells pre‐ and post‐inducible differentiation were harvested and inoculated into a 24‐well plate coated with Matrigel, each well 2 × 10^5^/500 μl and set three complex wells. Triplicate wells were cultured for 6 hrs, and photographs were obtained from three random fields of view. Numbers of nodes and rings were calculated using IMAGE‐PRO PLUS (MediaCybernetics, Shanghai, China) 6.0 software.

### Identification and suppression of the Notch signalling pathway *in vitro*

#### Treatment with VEGF165 and DAPT at different concentrations

Third passage rHFSCs were inoculated into a six‐well plate, and step‐wise *in vitro* inducible differentiation and suppression treatment were initiated when cells in the eighth day. For the induction group, KSR was replaced with FBS and EGF was removed in the culture medium. VEGF165 at a working concentration of 10 ng/ml was added for induction. For the suppression group, different concentrations of DAPT (0.5 and 1.0 μmol/l) were added based on the basic composition of the inducible medium. Cells in the two groups were cultured in triplicate at 37°C/5% CO_2_ for 1 week with medium changes every 2 days. Changes in cell morphology were examined using an inverted phase contrast microscope.

#### Western blot assays on associated proteins

After 1 week of induction, cell expression profiles for CD31 and VE‐cadherin in both the induction and suppression groups were examined by Western blot analysis. Cells were lysed on ice, the total protein was extracted, and the protein concentration was determined by the bicinchoninic acid method. SDS‐PAGE electrophoresis was then carried out, and proteins were transferred onto a film. Blots were then incubated with appropriate primary and secondary antibodies prior to chemiluminescence (colour development induced by ECLA/ECLB hybrid reagents) and photographing; Alpha software gel image analysis was used to determine expression profiles of CD31 and VE‐cadherin, which were compared based on optical density.

#### RT‐qPCR assays on associated RNA

After 1 week of induction, the RT‐qPCR assay was conducted on both the induction and inhibition groups. Two target genes (CD31, VE‐cadherin) of VECs and one internal reference gene (GAPDH) were amplified in a reaction tube. Primers were designed using Nisann Biological Technology Inc., (Shanghai, China) 5.0 software, and the primer information is presented in Table 1. Expression levels of ECs‐specific markers (CD31 and VE‐cadherin) were identified by isolating the total RNA from cells using the RNeasy total RNA isolation kit, reverse transcription for cDNA, the PCR reaction included 12.5 μl of 2 × qPCR Mix, 2 μl Primer Mix, 2.5 μl template and 8 μl ultrapure water. The relative expression of mRNA for each gene was measured using the ▵Ct method:▵Ct = target gene cycle threshold value ‐ reference gene cycle threshold value.

#### Dil‐ac‐LDL phagocytosis assay

After 1 week of induction, the Dil‐ac‐LDL phagocytosis assay was conducted on both the induction and suppression groups. Solutions at a working concentration of 10 μg/ml were prepared using complete medium and were added to six‐well plates containing rHFSCs groups. Cells were incubated for 4 hrs within a 37°C/5% CO_2_ incubator. The medium containing Dil‐ao‐LDL was removed, and the cells were washed three times with the medium‐free Dil‐ao‐LDL and PBS. Phagocytosis in each group was examined under an inverted fluorescence microscope.

### Preparations of rHFSCs carrying GFP virus *in vitro* and Matrigel mixture

GFP adenovirus and polybrene solution (both at a concentration of 1:1000) were added to the original medium for further use. Third passage rHFSCs in logarithmic growth were digested, suspended and cultured overnight in a 60‐cm plate. After reaching approximately 70–80% confluence, the medium was removed and changed to medium containing the virus. After homogenization, the culture was transferred into an incubator for further treatment. After approximately 24 hrs, the medium was changed to fresh medium and the infection efficiency was examined by fluorescence microscopy after 48 hrs. With infection stabilized, the rHFSC^GFP^ cells were digested and centrifuged. Matrigel mixtures were prepared on ice for subsequent use: Group A: 50 μl PBS + 250 μl Matrigel; Group B: 50 μl 10 ng/ml VEGF165 + 250 μl Matrigel; Group C: 50 μl 10 ng/ml VEGF165 (rHFSCs^GFP^) + 250 μl Matrigel. rHFSCs^GFP^ cells (2.0 × 10^6^) were harvested from each nude mouse.

### *in vivo* identification and vascularization of endothelial cells following induction from rHFSCs

#### Animals and study design

This work was conducted in compliance with applicable regulations for animal protection and ethics. Six‐week‐old male nude mice were injected intraperitoneally with 1% pentobarbital, and 300 μl of Matrigel mixture was injected subcutaneously into the abdomen of nude mice in groups A, B and C. Animals were fed a normal diet and sacrificed after 2 weeks. The Matrigel was removed for further observation and assaying.

#### Histological examination

Freshly collected specimens were fixed with 4% PFA solution for no less than 24 hrs. After dehydration and paraffin embedding, specimens were sectioned at 4 μm thickness. Paraffin sections were dewaxed in water and stained with haematoxylin–eosin. After being dehydrated and mounted, samples were examined and photographed under an inverted fluorescence microscope.

#### Immunohistochemical assays

CD31 expression in the tissue samples was determined as follows. Tissues were fixed, paraffin embedded and sectioned as described above. After being dewaxed, antigen retrieval was conducted using the hot retrieval method with 0.01 M citrate buffer for 15 min. After blocking with 8% BSA, the preparation was incubated with CD31 (1:100) primary antibody overnight at 4°C, followed by washing with PBS three times. Samples were then incubated with secondary antibody under ambient condition for 2 hrs and washed three times. DAB was added for 20 min. as the colour developing solution. After treatment with mounting agent, section was examined and photographed under a fluorescence microscope.

#### Immunofluorescence assay

Tissue samples were subjected to identical procedures to the frozen sections, including PBS washing, fixing in 4% PFA to remove endogenous peroxidase, BSA blocking, addition of CD31 (1:100) primary antibody and incubation at 4°C overnight. After three PBS washes, secondary antibody was added and samples were incubated under ambient condition. After PBS rinse and mounting, sections were examined and photographed under a fluorescence microscope.

#### Three‐dimensional reconstruction of blood vessels by rHFSCs^GFP^

After 2 weeks, anesthetized nude mice received intraperitoneal injection of 1% pentobarbital. Texas Red/dextran solution at a final concentration of 0.5 mg/ml was prepared and administrated retro‐orbitally at a dosage of 0.01 ml/g. After 15 min., the solution entered into the systemic circulation and target vasculature staining was achieved. Matrigel blocks were removed and observed under an upright two‐photon microscope (Olympus, Frankfurt, Germany). FV1000 drivers were used to acquire images, and Imaris software was used to determine 3D synthesis.

### Statistical analysis

Each experiment was repeated at least three times. The results are presented as the mean ± S.D. Comparisons between groups were performed by Wilcoxon test and *t*‐test. *P* < 0.05 was considered to indicate a statistically significant difference.

## Results

### Isolation, culture and purification of rHFSCs and identification results

#### Isolation, culture and purification of rHFSCs

**Figure 1:**
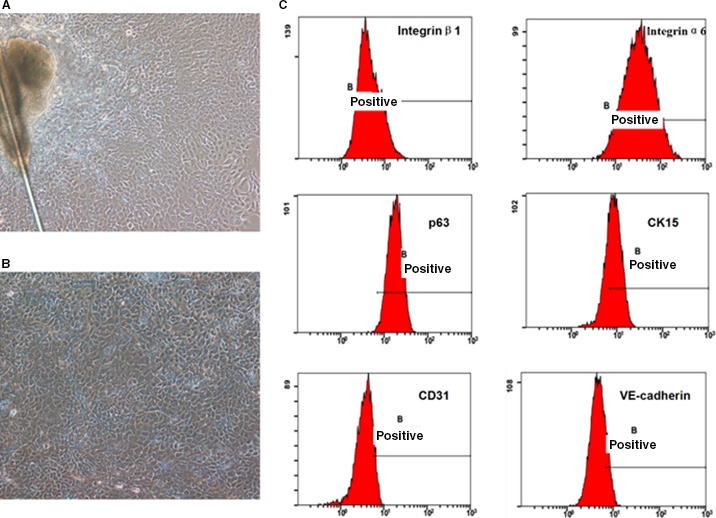
rHFSCs primary cells culture (×100) (**A**). The third‐generation cells after purified once (×100) (**B**) and flow cytometry detection of characteristic rHFSCs markers (**C**).

Under inverted phase contrast microscopy, cells began to disperse from the hair follicle bulge at approximately 2–3 days, with an increased cell numbers observed after 6–7 days when tightly packed cells showing a cobblestone‐like appearance and distribution. Third passage cells were purified based on morphology, following differential adherence to type IV collagen. Cell morphology was consistent with that of the primary cells, including typical cobblestone‐like appearance, good dioptre, strong cloning capability and typical biological characteristics of stem cells (Fig. 1A and B).

#### Flow cytometry

Characteristic markers of rHFSCs were assayed by flow cytometry. High expression levels of integrin‐β1 (98.87 ± 0.21%), integrin‐α6 (97.90 ± 0.33%) and P63 (98.50 ± 0.16%) were observed, with moderate expression of CK15 (66.87 ± 1.17%), and weak expression of CD31 (13.4 ± 0.26%) and VE‐cadherin (17.7 ± 0.18%). These results are consistent with those markers for rHFSCs (Fig. 1C).

#### Immunofluorescence

**Figure 2:**
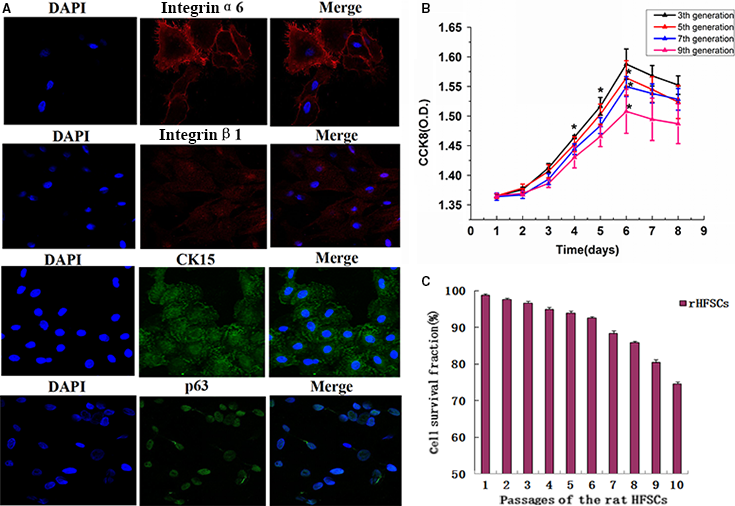
rHFSCs characteristic markers immunofluorescence staining (×63) (**A**). The growth curve of cells at different generations (**B**) and passages of the rHFSCs (**C**).

Immunofluorescence on rHFSCs identified the positive expression of integrin‐β1, integrin‐α6, CK15 and P63, and the negative expression of CD31 and VE‐cadherin. In accordance with these markers, these cells were identified as rHFSCs (Fig. 2A).

#### Cell proliferation and viability

As shown by the CCK‐8 cell proliferation assay, P3, P5, P7 and P9 cells were in a state of latency within the first 2 days, experienced clonal proliferation from day 3, and were in the logarithmic growth phase during days 4–6. Proliferation started to decrease from day 7, and the cells entered into the plateau phase from day 8. According to the S‐shaped growth curve, proliferation capability started to decrease from the P7 cells, with the P9 cells demonstrated even lower capabilities (Fig. 2B).

Cell viability using 0.4% trypan blue dye identified the passage 1–6 cells to be over 90% viable. They were 98.8% ± 0.40%, 97.63% ± 0.42%, 96.63% ± 0.45%, 94.93% ± 0.47%, 93.97% ± 0.57% and 92.57% ± 0.47%, respectively. The passage 7–9 cells were found to have slightly weaker activities, and they were 88.33% ± 0.75%, 85.87% ± 0.31% and 80.43% ± 0.75%, ranging from 80% to 90%. Moreover, the P10 cells demonstrated a significantly decreased cell viability, it was 74.60% ± 0.51%, but remained above 70% (Fig. 2C).

### Inducible differentiation of rHFSCs into endothelial cells *in vitro*

#### Flow cytometry on induced cells

**Figure 3:**
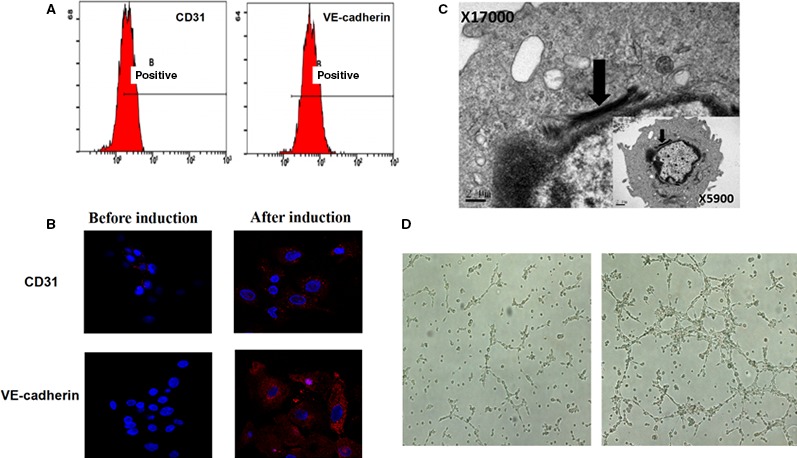
Flow cytometry detection of characteristic ECs markers (**A**), ECs characteristic markers immunofluorescence staining (×63) (**B**), observation of cell internal structure and W‐P corpuscle after induction (**C**) and the results of the two groups of cells bureaucratic form after 6 hrs (×40) (**D**).

Characteristic markers of VECs following 1 week of induction were investigated using flow cytometry. Moderate expression of CD31 (64.2 ± 0.21%) and strong expression of VE‐cadherin (93.6 ± 0.28%) were observed. Marker expression levels were higher than those pre‐induction (Fig. 3A).

#### Immunofluorescence on inducible cells

Immunofluorescence staining on cells following induction for 1 week identified high expression levels of CD31 and VE‐cadherin. The potential of these cells to undergo differentiation into endothelial cells was further validated (Fig. 3B).

#### Weibel–Palade Bodies (WPBs) examined by TEM

TEM at ×5900 magnification identified a flat nucleus, relatively abundant nuclear chromatin, prominent nucleoli, multiple mitochondria, Golgi apparatus and rough endoplasmic reticulum inside the cytoplasm. At a ×17,000 magnification, significant black rod‐like substructures could be found, which were consistent with the structural characteristics of endothelial cells (Fig. 3C).

#### Endothelial cell tube formation assay

Taking both the flow cytometry and immunofluorescence data together, high efficiency of vascular endothelial cell differentiation was demonstrated after 1 week of induction. Thus, an endothelial cell tube formation assay was implemented pre‐ and post‐induction. Linear structures were observed in the Matrigel at 6 hrs pre‐induction, and more integral tubular network structures were found at 6 hrs post‐induction (Fig. 3D). Numbers of tubes and nodes in the Matrigel were statistically higher in the induction group, compared to that in the non‐induction group (*P* < 0.05; Table 2).

**Table 2:** Comparison of numbers of tubes and nodes in Matrigel between pre‐ and post‐induction groups ($\overset{¯}{x}$ ± s)

Group	No. of tubes	No. of nodes
Before induction	5.3 ± 1.12	3.4 ± 1.04
After induction	23.5 ± 1.32^1^	21.2 ± 1.54^1^

^1^ *P* < 0.05, compared with the control group.

### Suppression of the Notch signalling pathway *in vitro* and its identification

#### Western blot assays

**Figure 4:**
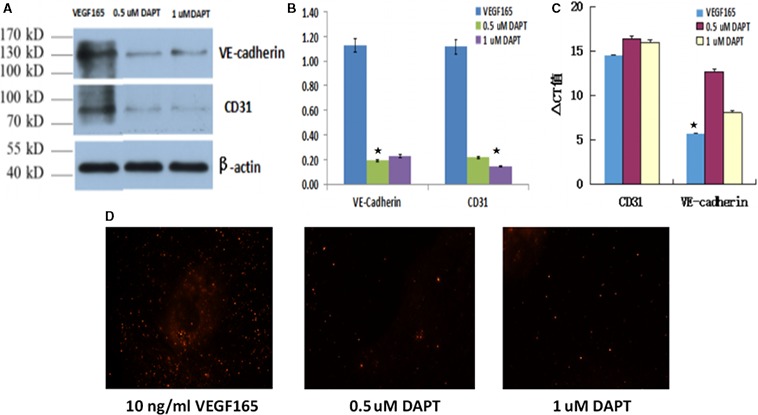
Protein expression of CD31 and VE‐cadherin in each group (**A**), grey value comparison of Western blot in each group (**B**), CD31 and VE‐cadherin mRNA levels comparison in each group (**C**) and Dil‐ac‐LDL phagocytosis assay in each group (×40) (**D**).

There were significant differences in the expression profiles of CD31 and VE‐cadherin, with induction group and two suppression groups demonstrating high and low expression, respectively. Minimal differences in the expression profiles of CD31 and VE‐cadherin were observed in the presence of DAPT at different concentrations (Fig. 4A and B).

#### RT‐qPCR assays

The relative expression levels of CD31 mRNA in induction group and two suppression groups after a week of inhibition are 14.47 ± 0.15, 16.42 ± 0.21 and 15.97 ± 0.28, respectively. The difference of ΔCt value of CD31 mRNA between the groups was not statistically significant (*P* > 0.05). The relative expression levels of VE‐cadherin mRNA, respectively, are 5.66 ± 0.25, 12.97 ± 0.13 and 8.08 ± 0.22. The difference of ΔCt value of VE‐cadherin mRNA between the groups was statistically significant (*P* < 0.05). However, all ΔCt values of VE‐cadherin mRNA were still in the high expression state. From VE‐cadherin mRNA level, we can see that the 0.5 μmol/l DAPT had a higher inhibitory effect than 1 μmol/l DAPT. However, the relative expression of CD31 between induction group and two suppression groups was not significantly different (*P* > 0.05; Fig. 4C).

#### Dil‐ac‐LDL phagocytosis assay

Phagocytosis of LDL is a key function of endothelial cells. The induction group demonstrated high phagocytosis capability of Dil‐ac‐LDL, with obvious fluorescent aggregation in the cytoplasm. In contrast, cells of the suppression group demonstrated almost no phagocytosis capability of Dil‐ac‐LDL at all concentrations of DAPT (Fig. 4D).

### Results of GFP adenovirus infection of rHFSCs *in vitro*

After 48 hrs, the infection rate of rHFSCs was found to be more than 90%. The target cells successfully labelled, as prerequisite for injection into the body, were used to investigate the role of the cells during the course of neovascularization and differentiation from host cells.

### *In vivo* experimentation

#### Gross observations

**Figure 5:**
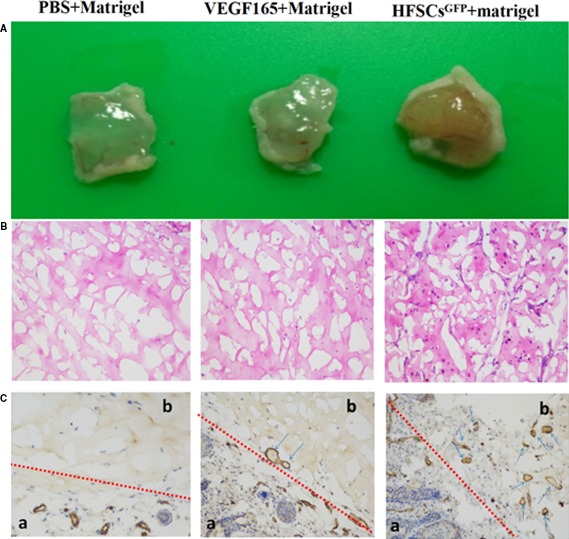
Gross appearance of three samples (**A**), and chemical testing of three Matrigel samples (On: HE staining, Below: CD31 immunohistochemistry; a: Epidermal area, b: Matrigel area) (**B** and **C**).

The nude mice were euthanized, and the Matrigel blocks were removed for analysis. Group A (PBS + Matrigel) had almost no blood vessel formation, with the presence of translucent colloids. Group B (VEGF165 + Matrigel) exhibited minimal capability to form blood vessels with sparse neovascularization. As expected, Group C (VEGF165 + rHFSCs^GFP^ + Matrigel) had comprehensive neovascularization, with an apparent visible network of blood vessels (Fig. 5A).

#### Histological and immunohistochemical assays

According to the HE staining and CD31 immunohistochemical staining findings, the potential of neovascularization in Group C was higher than that of groups A and B, with the appearance of mature and identifiable vessels. This finding suggested that the induced rHFSCs promote angiogenesis and have the capability to vascularize tissues, validating the *in vitro* experimental results (Fig. 5B and C).

#### Immunofluorescence

**Figure 6:**
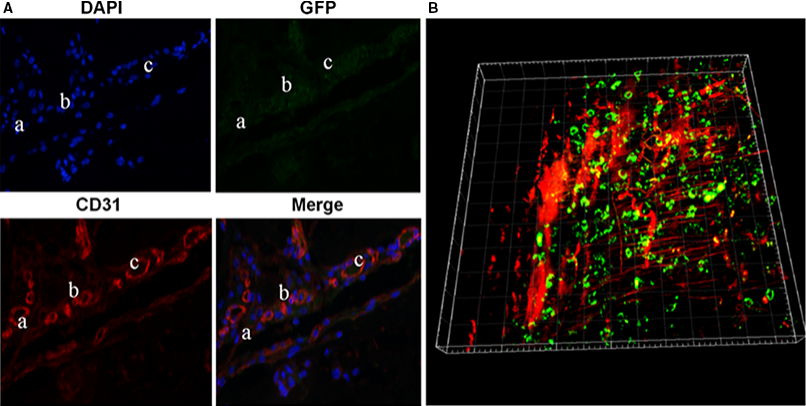
CD31 immunofluorescence of Matrigel in Group C (**A**), and three‐dimensional reconstruction of blood vessels and rHFSCsGFP in group C Matrigel sample (**B**).

To further demonstrate the role of induced rHFSCs during angiogenesis *in vivo*, Group C samples were immunostained for CD31. The results suggested that only a few HFSCs^GFP^ cells (green fluorescence) entered into the endothelial cell tube and expressed CD31 (red fluorescence), which is specific to endothelial cells. But it is rarely to see the co‐existence of both labels within the lumen. This result suggested that only a small population of induced rHFSCs was directly involved in the course of angiogenesis, through transverse differentiation into endothelial cells (Fig. 6A; parts a, b and c).

#### Three‐dimensional reconstruction of blood vessels and rHFSCs^GFP^

To further confirm the immunofluorescence results and more clearly demonstrate the role of induced rHFSCs during the course of angiogenesis, two‐photon microscopy equipped with 3D imaging was carried out. The Matrigel in Group C was found to have a rich and defined neovascularization network (red fluorescence), with HFSC^GFP^ cells clearly distributed in the peripheral blood vessels, and a small number of green fluorescent cells overlapping the vessels. This finding confirmed the immunofluorescence results, concluding that induced rHFSCs^GFP^ could promote early neovascularization. This promotion effect occurred through a small number of cells transversely differentiating into endothelial cells, while the majority of cells exerted their functions through accelerating host‐derived neovascularization (Fig. 6B).

## Discussion

HFSCs are a novel and emerging cell source, which could overcome the shortcomings of other stem cells owing to many advantages. At present, there are no authentic and specific markers for identification of HFSCs. Thus, it is now widely accepted that two or more markers are employed concurrently for their identification. Previous studies on HFSCs have demonstrated that higher expression profiles of integrin family members are associated with a high cell stemness and a low differentiation of target cells. Furthermore, integrin‐α6 and integrin‐β1 are well‐recognized surface markers of HFSCs and have been used concomitantly with other markers to identify these cells 42, 43. Another study found that 44 by HFSC‐specific markers K15 that combined with immunofluorescence labelling and flow cytometry, it would identify a high flux of HFSCs and a more accurate separation to other cells in a relatively short period of time. P63, a tumour suppressor, was also identified as a specific marker for epidermal stem cells. Because epidermal stem cells being present in the region of the hair follicle bulge, P63 was also proposed as an effective marker for the identification of HFSCs 45.Of course, there is no uniform medium used to culture the HFSCs, many researchers are still exploring. Furthermore, although the source of HFSCs is quite extensive, but its time to get primary cells is longer than fat stem cells or mesenchymal stem cells. With the further study, we believe that these problems could soon be resolved.

In this study, a modified microdissection technique was developed and employed. Furthermore, the procedure of mixed digestion of 1% type IV collagenase and 1% dispase, Matrigel‐coating, adherent culture of tissue blocks with gradient‐dosing and differential adhesion sorting of type IV collagen was followed. The resultant HFSCs demonstrated a typical cobblestone‐like appearance, good dioptre and strong cloning capability. Proliferation and viability assays using cells from distinct passages showed that the resultant cells underwent stages of latency, clonal proliferation, logarithmic growth and plateau. The growth curve was found to be S‐shaped, which is consistent with the growth characteristics of stem cells. With increased passage number, both proliferation and viability were decreased. However, trypan blue exclusion test suggested the viability of P1–6 cells remained at over 90%. The characteristic markers of rHFSCs assayed by flow cytometry and immunofluorescence included high expression of integrin‐β1, integrin‐α6 and P63, moderate expression of CK15, and weak expression of CD31 and VE‐cadherin. These results were consistent with those markers for rHFSCs. Highly homogenous HFSCs could be yielded through modified culture, amplification, purification and identification techniques from rHFSCs.

The induced differentiation of stem cells can be achieved by the following techniques: (*i*) chemicals and fluid; (*ii*) exogenous cytokines and proteins; (*iii*) transgene‐induced differentiation; and (*iv*) co‐culture with other cells and techniques. Distinct induction techniques can influence mature cell number, purity, maturity and survival rate of transplanted cells 46, 47, 48, 49. Based on these considerations, VEGF165 was found to be the most potent approach to induce HFSCs to differentiate into endothelial cells.

To determine whether the HFSCs were successfully induced to differentiate into endothelial cells, flow cytometry, immunofluorescence, endothelial cell tube formation assay and TEM were applied in combination. CD31 (platelet endothelial cell adhesion molecule‐1) and VE‐cadherin (vascular endothelial cadherin) were found to be predominantly expressed at the plasma membrane and cytoplasm. Both factors are considered to be specific markers for the identification of endothelial cells and can be used to identify, sort and isolate these cells through flow cytometry and immunofluorescence approaches. Regarding endothelial regeneration, it is critical to maintain endothelial integrity and vascular tone 39, 40, 50. In this study, after induction of VEGF165 (10 ng/ml) for 1 week in culture, there were notable increases in the expression profiles of CD31 and VE‐cadherin, as well as their titres, as shown by flow cytometry and immunofluorescence. In 1982, Wagner *et al* discovered WPBs using TEM. These organelles were found to be unique to endothelial cells and were considered to be the most characteristic parameter. The abundance of endothelial cell tube formation is also generally considered to be a characteristic of endothelial cells, phagocytic cells and some tumour stem cells. To further confirm the functional and structural integrity of the endothelial cells induced in this study, the WPBs of cells induced by 10 ng/ml of VEGF165 were examined by endothelial cell tube formation under TEM. After 6 hrs, cell tube formation was demonstrated and the numbers of tubes and nodes were statistically higher in the induction group than in the non‐induction group (*P* < 0.05). Furthermore, WPBs could be detected after induction. The relatively high induction efficacy using this technique was demonstrated through multiple approaches, indicating that the cells differentiated into endothelial cells in terms of structure, function and protein expression.

To verify the ability of rHFSCs to form neovascular structures after induction *in vitro* and to clarify their pro‐angiogenic ability *in vivo*, Matrigel was used to coat the cells prior to administration *in vivo*. After 2 weeks, all Matrigel blocks were removed from the nude mice. HE staining and CD31 immunohistochemical staining found the level of CD31^+^ expression was highest in Group C as compared to groups A and B, with the appearance of mature and comprehensive vessels. This finding suggested that the induced rHFSCs promote angiogenesis and have the capability to vascularized tissues, validating the *in vitro* experiments. Immunofluorescence and 3D reconstruction of blood vessels were employed to depict the spatial relationship between the induced rHFSCs and the angiogenesis. The results demonstrated that in the Matrigel (Group C), few HFSCs^GFP^ cells (green fluorescence) were found in the endothelial cell tube, with the majority distributed in the peripheral blood vessels. Furthermore, rich neovascularization and a definite vascular network (red fluorescence) were observed. It can be concluded that the induced rHFSCs^GFP^ promoted early neovascularization. This promotion effect occurred through some transverse differentiation into endothelial cells, but mostly through promotion of host‐derived neovascularization. Some studies have shown that 51, 52 MSCs could be differentiate into some CD31^+^ endothelial cells *in vivo*, then Promoted angiogenesis, increase blood flow of Mice with chronic ischaemia limb. And the role of ASCs early after aspirated fat transplantation may be to induce new vessels from the recipient region to grow around and into the graft by releasing significant amounts of angiogenic growth factors rather than to differentiate into ECs, pericytes or smooth muscular cells forming new vessels, an effect that might be enhanced by hypoxia 53. According to the above,structures of blood vessels: multilayered, hollow tube. Inner layer: VECs, stroma membrane; middle layer: smooth muscle, elastic fibres, collagen fibres; Outer layer: the connective tissue. Although it can be more successful induced by endothelial cells with good performance *in vitro*, the evolution is more complex in the body, to make it capable of forming new blood vessels, or directly involved in hosting large horizontal differentiation of new blood vessels in the intimal layer needs more exploration, and this will be the next phase of our group focuses on. Current results show that these cells accelerated angiogenesis in host in the body after inducing, and paracrine may play a major role in this evolution.

To conclude, we have refined and optimized methods for the isolation, culture and purification of rHFSCs. This established system is relatively simple and effective to induce the differentiation of HFSCs into endothelial cells. By examining the role of the Notch signalling pathway during differentiation into endothelial cells, we elucidated the form and the efficiency of rHFSCs^GFP^ in the course of pro‐angiogenesis *in vivo*. This study provides a theoretical basis for blood vessel construction, angioplasty and early vascularization of wounds, as well as new insights into the use of tissue‐engineered skin for the clinical treatment of ischaemic diseases.

## Author's contributions

R‐F Quan conceived and designed the experiments and drafted the manuscript. R‐F Quan and X‐M Wu conceived and supervised the study. W‐B Du, X Ji and R‐X Shao performed experiments, acquired data. W‐B Du, X Zheng and S‐C Xu analysed the data and revised the manuscript. R‐F Quan, X‐M Wu and D‐S Yang interpreted data and critically revised the manuscript. All authors read and approved the final version of the manuscript.

## Competing Financial interest

The authors declare no competing financial interests.
